# Molecular Mechanisms in Clear Cell Renal Cell Carcinoma: Role of miRNAs and Hypermethylated miRNA Genes in Crucial Oncogenic Pathways and Processes

**DOI:** 10.3389/fgene.2019.00320

**Published:** 2019-04-24

**Authors:** Eleonora A. Braga, Marina V. Fridman, Vitaly I. Loginov, Alexey A. Dmitriev, Sergey G. Morozov

**Affiliations:** ^1^Institute of General Pathology and Pathophysiology, Moscow, Russia; ^2^Vavilov Institute of General Genetics, Russian Academy of Sciences, Moscow, Russia; ^3^Research Center of Medical Genetics, Moscow, Russia; ^4^Engelhardt Institute of Molecular Biology, Russian Academy of Sciences, Moscow, Russia

**Keywords:** clear cell renal cell carcinoma, microRNA, target genes, angiogenesis, epithelial-mesenchymal transition, metastasis, hypermethylated miRNA genes, long non-coding RNA

## Abstract

Clear cell renal cell carcinoma (ccRCC) is the third most common urological cancer, and it has the highest mortality rate. The increasing drug resistance of metastatic ccRCC has resulted in the search for new biomarkers. Epigenetic regulatory mechanisms, such as genome-wide DNA methylation and inhibition of protein translation by interaction of microRNA (miRNA) with its target messenger RNA (mRNA), are deeply involved in the pathogenesis of human cancers, including ccRCC, and may be used in its diagnosis and prognosis. Here, we review oncogenic and oncosuppressive miRNAs, their putative target genes, and the crucial pathways they are involved in. The contradictory behavior of a number of miRNAs, such as suppressive and anti-metastatic miRNAs with oncogenic potential (for example, miR-99a, miR-106a, miR-125b, miR-144, miR-203, miR-378), is examined. miRNAs that contribute mostly to important pathways and processes in ccRCC, for instance, PI3K/AKT/mTOR, Wnt-β, histone modification, and chromatin remodeling, are discussed in detail. We also separately consider their participation in crucial oncogenic processes, such as hypoxia and angiogenesis, metastasis, and epithelial-mesenchymal transition (EMT). The review also considers the interactions of long non-coding RNAs (lncRNAs) and miRNAs of significance in ccRCC. Recent advances in the understanding of the role of hypermethylated miRNA genes in ccRCC and their usefulness as biomarkers are reviewed based on our own data and those available in the literature. Finally, new data and perspectives concerning the clinical applications of miRNAs in the diagnosis, prognosis, and treatment of ccRCC are discussed.

## Introduction

Renal cell carcinoma (RCC) is diagnosed in approximately 90% of patients with kidney cancer. RCC has the highest mortality rate among urogenital cancers (Vasudev et al., 2012). Approximately 270,000 new cases of RCC and 116,000 RCC-related deaths are reported globally every year (Randall et al., 2014). RCC is a heterogeneous group of epithelial tumors, among which clear cell RCC (ccRCC) is the most common and accounts for 70–80% of the reported cases of RCC (Cairns, 2010). The severity of ccRCC is higher than that of papillary kidney cancer and chromophobe tumors (Cairns, 2010). Lack of effective diagnostics in the early stages of the disease, increasing mortality rate, and resistance to therapies in patients with metastatic ccRCC emphasize the need to discover new biomarkers that are applicable for the early diagnosis of ccRCC and detection of metastasis.

Epigenetic regulatory mechanisms involving DNA methylation at the genomic transcriptional level and the interaction of non-coding RNAs, in particular microRNAs (miRNAs), with target messenger RNA (mRNA) at the post-transcriptional level are important in the regulation of genes and proteins (Baylin and Jones, 2016). miRNAs are involved in the regulation of cell proliferation, differentiation, and stress responses, and in the regulation of other fundamental biological processes and signaling pathways (Cora et al., 2017). Alterations in expression and regulatory functions of miRNA can be one of the key factors of various pathogeneses. miRNAs are involved in the development of more than 300 diseases, including oncological diseases. The number of publications aimed at identifying target genes and signaling pathways that involve miRNAs has increased (Reddy, 2015; Dragomir et al., 2018; Gattolliat et al., 2018). miRNAs are important positive or negative regulators of all processes characteristic of the pathogenesis of tumors, which include control of the cell cycle, apoptosis, neo-angiogenesis, tissue invasion, and metastasis (Exposito-Villen et al., 2018; Goradel et al., 2018; Kashyap et al., 2018; Mens and Ghanbari, 2018).

miRNAs are classified as being oncogenic or oncosuppressive (tumor suppressive) based on their stimulating or inhibiting effects, respectively, on tumor development. The targets of oncogenic miRNAs usually include the mRNAs of the tumor suppressor genes. In contrast, the targets of miRNAs that act to suppress tumor development are oncogenes and genes involved in tumor progression. The regulation of tumor suppressor miRNAs involves methylation of the promoter regions of their genes. The methylation suppresses their expression and subsequently inhibits their suppressor function. These processes occur more often in genes encoding tumor suppressor miRNAs than in genes encoding tumor suppressor proteins (Kunej et al., 2011; Piletic and Kunej, 2016). Methylation of tumor suppressor miRNA genes and the interaction of miRNAs with target mRNAs have a systemic effect on key processes and signaling pathways involved in carcinogenesis (Lopez-Serra and Esteller, 2012; Loginov et al., 2015; Baylin and Jones, 2016). Studies of methylation and profiling of miRNA expression have driven the design of minimally invasive diagnostics, and recent advances in the field of cancer epigenomics have highlighted the profound possibilities of these approaches in clinical practice (Li et al., 2015; Xing and He, 2016; Morris and Latif, 2017).

Epigenetic mechanisms involved in ccRCC genesis have received less attention than other cancers, such as colorectal, lung, breast, and prostate carcinomas. Nevertheless, many genes susceptible to hypermethylation have been identified, including *VHL, RASSF1A, CDH1*, and *APAF1*, and reported as promising biomarkers of ccRCC (for example, Dmitriev et al., 2014; Braga et al., 2015; Xing and He, 2016; Morris and Latif, 2017). Data on the expression profiles of miRNAs, their target genes in ccRCC, and their potential use in the clinic are accumulating and have been covered in earlier reviews (for example, Li et al., 2015; He Y.H. et al., 2018). However, the contribution of methylation of miRNA genes to epigenetic regulatory mechanisms in ccRCC remains unclear, although interesting results have been reported.

This review examines the expression profiles, targets, and functions of oncogenic and tumor suppressor miRNAs, their role in molecular mechanisms and signaling pathways of ccRCC, as well as their clinical potential. In addition, based on data obtained in our studies and the published literature, we consider in detail the advances made in studies of hypermethylated miRNA genes in ccRCC and their usefulness as biomarkers.

Several long non-coding RNAs (lncRNAs) have been recently described in RCC, as has their involvement in regulatory interactions as competing endogenous RNA (ceRNA), whereby lncRNAs can act as miRNA sponges to modulate gene expression (Li et al., 2017; Chan and Tay, 2018). Moreover, integrative analyses have enabled construction of a ccRCC regulatory ceRNA network comprising 89 lncRNAs, 10 miRNAs, and 22 mRNAs (Yin et al., 2018). In general, however, very few publications have addressed lncRNAs in relation to ccRCC (60 papers on the topic were found indexed in PubMed as of January 2019). In this review, we also consider some data on lncRNAs and their interactions with miRNAs that are relevant to ccRCC.

## Oncogenic and Suppressive miRNAs in Main Signaling Pathways of ccRCC

Expression microchips and quantitative PCR have been used to identify miRNAs with decreased expression levels in kidney tumors, presumably oncosuppressor miRNAs, and with increased expression levels in ccRCC, presumably oncogenic miRNAs (for example, Li et al., 2015). The expression of the miRNA genes is generally evaluated by determining the levels of mature miRNA, which is very stable and thus an effective biomarker (Andorfer et al., 2011).

Tumor suppressor miRNAs include miR-34a (Yamamura et al., 2012; Zhang C. et al., 2014; Toraih et al., 2017), miR-30c (Huang J. et al., 2013), miR-30d (Wu et al., 2013), miR-99a (Cui et al., 2012), miR-133a (Kawakami et al., 2012), miR-133b (Wu et al., 2014), miR-138 (Ding et al., 2018), miR-141 (Li W. et al., 2014), miR-143 (Yoshino et al., 2013), miR-182 (Kulkarni et al., 2018), miR-187 (Zhao et al., 2013), miR-199a (Qin et al., 2018), miR-200c (Ding et al., 2018), miR-205 (Xu et al., 2018), and others. These miRNAs target mRNAs of oncogenes or genes encoding proteins mediating the progression of kidney tumors. For example, the targets of the crucially important miR-34a are *c-MET* (Toraih et al., 2017), *c-MYC* (Yamamura et al., 2012), and *NOTCH1* (Zhang C. et al., 2014) oncogenes. All are involved in the proliferation and activation of the cell cycle. Many targets of suppressor miRNAs are associated with invasion and migration. For example, matrix metalloproteinase 9 (MMP-9) is a validated target of miR-133b (Wu et al., 2014). *VIM, EZH2, ZEB2* (Ding et al., 2018), and *HIF1A* (Song et al., 2011) are regulated by miR-138.

Typical oncogenic miRNAs include miR-7 (He H. et al., 2018), miR-21 (Li X. et al., 2014; Chen J. et al., 2017; Cui et al., 2017), miR-155 (Ji et al., 2017), miR-590-5p (Xiao et al., 2013), and others. Their targets, on the contrary, are tumor suppressor genes, which include *PDCD4* (Li X. et al., 2014), *PTEN* (Cui et al., 2017), *TIMP3* (Chen J. et al., 2017), *FOXO3A* (Ji et al., 2017), *PBRM1* (Xiao et al., 2013), non-coding *MEG3* (He H. et al., 2018), and others.

Modern methodologies have provided a great deal of information concerning the miRNA expression profiles in ccRCC (He H. et al., 2015; Wotschofsky et al., 2016). Sixty-three differentially expressed miRNAs have been identified by analyzing the massive sequencing data published in The Cancer Genome Atlas (Liang et al., 2017).

To date, a substantial amount of data has been obtained on the role of miRNA in the regulation of target genes in ccRCC and its pathogenesis (Jung et al., 2009; Redova et al., 2011; Heinzelmann et al., 2014). A study of miRNA and mRNA gene networks constructed on the basis of their expression profiles in ccRCC reported a key role for miR-106a-5p. The loss of this miRNA led to the increased expression of 49 putative targets (Müller and Nowak, 2014). Other miRNAs implicated in this study were miR-135a-5p (32 targets), miR-206 (28 targets), miR-363-3p (22 targets), and miR-216b (13 targets) (Müller and Nowak, 2014). The targets included genes that affect apoptosis, metastasis, cellular mobility, and oncogenes (*c-MET, VEGFA, NRP2*, and *FLT1*). A similar study (Butz et al., 2015) pinpointed miR-124-3p, miR-30a-5p, and miR-200c-3p as the most significant miRNAs affecting protein expression in ccRCC; the expressions of these miRNAs were often reduced.

Notably, since the von Hippel Lindau (*VHL*) gene has an important role in both familial and sporadic kidney cancer, the genes associated with VHL-dependent regulation are important candidates in studying the spectrum of miRNAs that alter *VHL* expression (Neal et al., 2010). Loss of VHL function results in constitutive activation of the hypoxia-inducible factor (HIF) pathway, which leads to hypoxia and subsequent expression of angiogenic factors. Oncogenic miRNAs that enhance the development of hypoxia and angiogenesis and their targets have been identified. The next section of this review is devoted to a detailed look at the VHL/HIF pathways and the involved miRNAs.

The significance of the phosphoinositide 3-kinase (PI3K)/protein kinase B (AKT)/mechanistic target of rapamycin (mTOR) pathway (Joosten et al., 2018) associated with the escape from apoptosis, growth, and proliferation of cells in kidney cancer must be noted. The Cancer Genome Atlas Research Network indicates frequent mutations in the genes of this pathway in ccRCC. The PI3K/AKT/mTOR pathway can also stimulate HIF through mTOR modulation (Cancer Genome Atlas Research, 2013).

miR-148a targets *AKT2*, and this interaction leads to the suppression of cell growth, colony formation, migration, and invasion and tumor growth in xenografts. The miR-148a level is decreased in RCC and is inversely correlated with tumor size, stage, and metastasis to lymph nodes (Cao et al., 2017). In cultured RCC cells, the inhibition of *FLOT1* by miR-182-5p reduces the phosphorylation and activation of AKT2 and subsequent phosphorylation of FOXO3a (Xu et al., 2014). Oncosuppressor and transcription factor FOXO3A is translocated out of the nucleus upon phosphorylation by proteins such as Akt (protein kinase B). FOXO3a decreases proliferation and arrests cells in the G1 phase. The level of miR-182-5p is often reduced in RCC (Xu et al., 2014).

On the contrary, miR-122 mimic increases the level of phosphorylation of AKT2 and mTOR. In addition to its enhanced expression in RCC, miR-122 reduces the expression of the Sprouty RTK signaling antagonist 2 (*SPRY2*) gene. The product of this gene is the inhibitor of Ras/MAPK signaling pathway (Wang Z. et al., 2017). Thus, while miR-148a and miR-182-5p act as tumor suppressors (Fan Y. et al., 2016; Cao et al., 2017), miR-122 acts as an oncogene (Lian et al., 2013).

The expression of miR-99a was first shown to be downregulated in RCC tumor tissues and correlated with poor survival (Cui et al., 2012). The authors also reported that the use of its mimic suppressed cell growth, migration, invasion *in vitro* and *in vivo*, and induced cell arrest at the G1 phase (Cui et al., 2012). However, in contrast to these results, which indicated tumor suppression activity of miR-99a, a recent study (Oliveira et al., 2017) described the upregulation of miR-99a and downregulation of its target gene mTOR in most of the ccRCC samples examined. These findings indicated the oncogenic activity of miR-99a. Both studies implicated mTOR as the direct target of miR-99a in renal cancer cells. Further studies are needed to definitively determine the suppressive or oncogenic activity of miR-99a in ccRCC.

miR-144 also targets *mTOR* in RCC. The reduced expression of miR-144 has been inversely correlated with the stage and size of the tumor, while increased miR-144 expression can suppress cell growth and arrest cells in the G1 phase (Xiang et al., 2016). However, another study (Xiao et al., 2017) identified miR-144-3p as an oncomiRNA. The authors described that the overexpression of miR-144-3p promoted proliferation, migration, invasion, and chemoresistance in ccRCC cells. They also identified AT-rich interactive domain 1A (*ARID1A*) as a direct target gene of miR-144-3p in ccRCC. ARID1A is a transcription regulator that is a part of the SNF/SWI remodeling complex. The data to date highlight the lack of clarity concerning the role of miR-144 in ccRCC. Further in-depth studies are required.

miR-137 expression is reduced in RCC and miR-137 suppresses the activation of the PI3K/AKT signaling pathway. This leads to reduced proliferation, migration, and invasiveness of cells, enhanced apoptosis, and suppressed tumor growth in xenografts (Zhang and Li, 2016). The lncRNA *SNHG1* also negatively regulates this miRNA in RCC (Zhao S. et al., 2018).

The target of most oncogenic miRNAs in ccRCC is the tumor suppressor phosphate and tensin homolog (*PTEN*) gene, which is the negative regulator of the PI3K/AKT/mTOR pathway. In particular, *PTEN* directly interacts with miR-23b (Zaman et al., 2012), miR-193-3p (Liu L. et al., 2017), miR-21 (Cui et al., 2017), and miR-22 (Fan W. et al., 2016), although miR-22 acts as a tumor suppressor. Appropriate interactions that affect the expression of PTEN may be significant in the development of the disease. Aggressive ccRCC specimens are characterized by a reduced level of PTEN (Cancer Genome Atlas Research, 2013). There are some indications that miR-193a-3p and miR-224 can affect the PI3K/AKT pathway by targeting the ST3 beta-galactoside alpha-2,3-sialyltransferase 4 (*ST3GalIV*) gene in RCC (Pan et al., 2018a).

**FIGURE 1:**
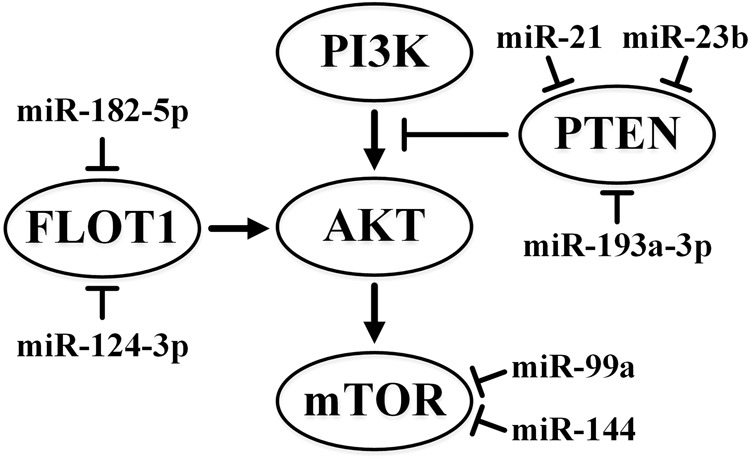
Summary of the effects of miR-21, miR-23b, miR-99a, miR-124-3p, miR-144, miR-182-5p, and miR-193a-3p on the PI3K/AKT pathway in ccRCC. ↑ indicates activity stimulation and ⊤ indicates activity suppression according to any of the mechanisms.

The influence of some miRNAs on the PI3K/AKT pathway is depicted in Figure 1.

It is believed that the effect of the reduced expression of miR-124-3p in ccRCC is due to an increase in the expression of its putative targets, *CAV1* and *FLOT1*, which are cytoplasmic membrane proteins associated with caveolae-mediated endocytosis (Butz et al., 2015). CAV1 expression causes the AKT-dependent formation of lamellipodia, which increases cell migration and invasion. In addition, it activates the Ras-extracellular signal-regulated kinase (Ras-ERK) pathway and suppresses anoikis, a form of programmed cell death specific to non-malignant cells induced by the detachment of anchorage-dependent cells. FLOT1 is also associated with endocytosis and activation of epidermal growth factor receptor, and subsequent activation of MAPK (Butz et al., 2015).

Studies have also shown the importance of the Wnt/β-catenin signaling pathway in RCC and indicated that many WNT antagonist genes are inhibited by methylation of their promoters in ccRCC (Joosten et al., 2018). In ccRCC, miR-106b-5p can activate this pathway, having as its targets the following negative regulators of the Wnt/β-catenin pathway: *LZTFL1, SFRP1*, and *DKK2* (Lu et al., 2017). Thus, being an oncogene, miR-106b-5p supports the aggressiveness and stemness phenotypes of ccRCC (Lu et al., 2017). miR-1260b also targets *DKK2* and stimulates the proliferation and invasiveness of RCC cells, and its expression is enhanced in RCC (Hirata et al., 2013).

miR-203a targets the glycogen synthase kinase-3β (*GSK3β*) gene (Hu et al., 2014), whose product is involved in β-catenin degradation (Joosten et al., 2018). Thus, miR-203a also acts as an oncogene in RCC, being a predictor of poor prognosis (Hu et al., 2014). IGF-II mRNA binding protein 1 (IGF2BP1), which participates in the Wnt/β-catenin pathway, is a target of miR-372 in the RCC cell lines, thus miR-372 acts as a tumor suppressor (Huang et al., 2015).

The roles of miRNAs in important processes in ccRCC, which include cell migration, invasion, and proliferation, were highlighted in a recent review (He Y.H. et al., 2018).

Notably, mutations in known oncogenes and tumor suppressors, such as *RAS, TP53, RB*, and *PTEN*, are not typical of RCC (Joosten et al., 2018). However, sporadic mutations occur with high frequency in the genes encoding proteins associated with histone modification and chromatin remodeling. These genes include *SETD2* (3–12%), *KDM5C* (3–8%), *KDM6A* (1%), *BAP1* (8–15%), and *PBRM1* (21–41%) (Cancer Genome Atlas Research, 2013). miRNA-mediated repression also contributes to the altered expression of these genes in RCC. For example, miR-106a-5p, which targets *SETD2*, functions as an oncogene in this case. SETD2 is instrumental in suppressing and halting the cell cycle at the transition from G0 to G1, and also inhibits proliferation and stimulates apoptosis in RCC (Xiang et al., 2015). SETD2 increases the binding of H3K36me3 to the p53 promoter that enhances the expression of p53. In ccRCC, miR-590-5p acts as an oncogene and its *PBRM1* target acts as a tumor suppressor (Xiao et al., 2013).

Immune checkpoint molecules play an important role in the control of carcinogenesis. In ccRCC, the B7 homolog 3 (*B7-H3*) immune checkpoint molecules are validated targets of miR-187. This miRNA exhibits the characteristics of a tumor suppressor, and its concentration is reduced in both tumor tissue and plasma that provides opportunities for use in diagnosis of patients with ccRCC. A low level of miR-187 is associated with advanced stages of the disease and a worse 5-year survival rate. *In vitro*, it reduces cell proliferation and mobility and suppresses tumor growth (Zhao et al., 2013). Non-classical human leukocyte antigen G (HLA-G) inhibits the cytotoxic activity of T-lymphocytes and natural killer cells. *HLA-G* is often expressed in RCC and is a target of miR-148a and miR-133a, and overexpression of these miRNAs suppresses its effect (Jasinski-Bergner et al., 2015).

**FIGURE 2:**
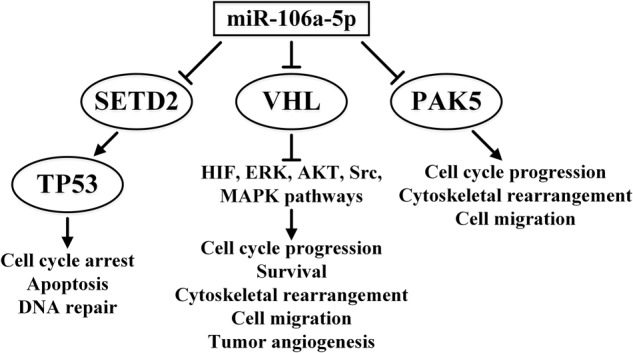
Multiple features of the effects of miR-106a-5p in ccRCC signaling pathways. The overwhelming effect of miR-106a-5p on tumor suppressors TP53 and VHL and oncogene PAK5 is presented. ↑ indicates activity stimulation and ⊤ indicates activity suppression according to any of the mechanisms.

It is interesting to note the dual functions of some miRNAs in ccRCC. For instance, one of the targets of miR-106a-5p is the P21 (RAC1) Activated Kinase 5 (*PAK5*) gene. PAK5 is a serine/threonine kinase (activated by RAC) that regulates cytoskeleton dynamics, cell proliferation, and survival. miR-106a-5p is reportedly a tumor suppressor that reduces metastasis in xenografts and reduces migration and invasiveness of RCC cell lines (Pan et al., 2017). These data contradict the studies that reported oncogenic effects of miR-106a-5p, such as its ability to target *SETD2* and indirectly downregulate TP53 (Xiang et al., 2015) and VHL protein levels (Oliveira et al., 2017). The contradictory nature of the influence of miR-106a-5p on ccRCC is summarized in Figure 2.

Thus, some miRNAs (for example, miR-106a and miR-144) can show both tumor suppressor and oncogenic properties depending on the context, i.e., their effect is apparently dependent on the target gene and pathways involved. However, some contradictory data, for instance, for miR-99a (Cui et al., 2012; Oliveira et al., 2017), may be the result of inconvenient methodology or inadequate sampling of examined tumors.

The next few sections of this review will be devoted to discussing the regulation of angiogenesis and epithelial-mesenchymal transition (EMT), given the great importance of these processes in RCC.

## Molecular Mechanisms in Hypoxia and Angiogenesis in ccRCC: the Role of miRNA

In most clear cell carcinomas of the kidney, the expression of the *VHL* gene is dysregulated in a similar manner (98% of cases feature gene deletion, with hypermethylation and point mutations in the remaining 2%). VHL binds HIF1A and degrades it only in the presence of oxygen. In the absence of oxygen, HIF1A dimerizes with HIF1B and leads to the activation of transcription of more than 40 genes, including the vascular endothelial growth factor (*VEGF*) family and erythropoietin (*EPO*) genes, genes encoding growth factors (e.g., *EGF, PDGF*) and glycolytic enzymes, and genes involved in glucose metabolism (e.g., *GLUT1/4*) (Roskoski, 2017). In the presence of *VHL* mutations downregulating its expression, the aforementioned regulatory influences of *VHL* on HIF1 are disrupted. Inactivation by oxygen is also a characteristic of HIF2A (Serocki et al., 2018). The VEGF-A protein of the VEGF family stimulates the dimerization and activation of the VEGF receptor 2 (VEGFR2). This interaction regulates, in particular, the angiogenesis process, ensuring the supply of oxygen and nutrients to the tumor and encouraging metastasis. The activation of VEGFR2, in turn, leads to the activations of the ERK1/2, AKT, Src, and p38 MAPK pathways, which are important for tumor development and metastasis. These activations occur through a cascade of phosphorylation events involving the tyrosine moiety of various proteins, which further recruit phosphotyrosine-binding proteins. These pathways are associated with key angiogenesis events that include cell proliferation, migration, survival, and vascular permeability (Roskoski, 2017). In patients with ccRCC being treated with VEGF and mTOR blockers, angiogenesis can be stimulated by angiopoietin 2, c-MET, or interleukin (Malouf et al., 2016).

While HIF1 responds to acute hypoxia, the adaptation to chronic hypoxia is mediated by the expression of HIF2 and HIF3 in endothelial tissues, while the expression of HIF1 is downregulated. miRNAs play a significant role in this adaptation. While there is a significant overlap of HIF1 and HIF2 targets, the role of HIF3 is much less clear. It has at least six isoforms. One suppresses the activity of HIF1 and HIF2, while others may activate the transcription of a number of genes (Serocki et al., 2018).

In response to prolonged hypoxia, 786-O, a VHL-defective cell line of ccRCC, displays upregulation of the expression of *L1CAM* and *FBN1* genes. In addition to the downregulated expression of miR-100 and miR-378, 786-O hypoxic cells have downregulated expression levels of *AUTS2, MAPT, AGT*, and *USH1C* genes. Expression of miR-100 and miR-378 was also found to be significantly reduced in bone metastases of ccRCC (Chen S.C. et al., 2017).

In ccRCC, the response to hypoxia and metastasis is also modulated by the androgen receptor (AR). AR increases the expression of miR-185-5p, which targets *VEGF-C* (a gene encoding lymphangiogenetic factor). At the same time, binding of miR-185-5p to the promoter region of *HIF2A* mRNA increases HIF2A expression and the subsequent expression of *VEGFA* (an angiogenetic factor). This explains why AR-positive ccRCC metastasizes to the lung rather more often than to lymph nodes (Huang et al., 2017).

In RCC patients, especially those diagnosed with ccRCC and VHL disorders caused by familial mutations, disruption of signaling pathways inhibited by tyrosine kinase inhibitors and other related inhibitory drugs is most effective (Mickley et al., 2015). The possibility of using drugs based on miRNA mimics and anti-miR is also being considered for these purposes (Schanza et al., 2017; Serocki et al., 2018).

As mentioned before, miRNAs contribute significantly to regulation of the main hypoxia response pathways. At least 40 miRNAs affect the expression of HIF alone in various tissues, including miR-687 in embryonic renal tissue and miR-429 and miR-19a in endothelial cells (Serocki et al., 2018). Upregulation of miR-106a and miR-106b and downregulation of their target gene *VHL* has been described for ccRCC (Oliveira et al., 2017).

*VEGFR2* is the putative target of miR-221 in metastatic RCC (Khella H.W.Z. et al., 2015). Accordingly, poor survival outcome in patients treated with the receptor tyrosine kinase inhibitor sunitinib correlates with a high level of miR-221 accompanied by low levels of VEGFR2. The effect of miR-221 is different for endothelial and tumor cells; it lowers proliferation and angiogenesis in human umbilical vein endothelial cells (HUVECs) but increases the proliferation of ACHN kidney cancer cells (Khella H.W.Z. et al., 2015).

Responses to hypoxia are also influenced by lncRNA. Antisense transcripts of the 3′ and 5′ non-coding regions of *HIF1A* are 3′aHIF-1α and 5′aHIF-1α, respectively. In human lung epithelial cells, the expression of these lncRNAs is induced by hypoxia, and they can suppress the expression of *HIF1A* (Uchida et al., 2004). In addition, increased expression of the HOX transcript antisense intergenic RNA (HOTAIR) lncRNA is observed in RCC, and it correlates with the tumor progression. It has a direct inhibitory effect on miR-217, the target of which is *HIF1A*. Suppression of miR-217 reduces apoptosis, increases proliferation and migration of tumor cells, and stimulates EMT in RCC (Hong et al., 2017).

In RCC and ovarian cancer, miR-192 has anti-angiogenic properties due to the effect on its *EGR1* and *HOXB9* targets (Wu et al., 2016). In lung cancer, EGR1 binds to the *VEGFA* proximal promoter, activating its expression (Shimoyamada et al., 2010). The effect of HOXB9 on vascularization in breast cancer has been documented (Seki et al., 2012). Moreover, low levels of miR-192 in various malignant tumors are a predictor of poor prognosis (Wu et al., 2016).

**FIGURE 3:**
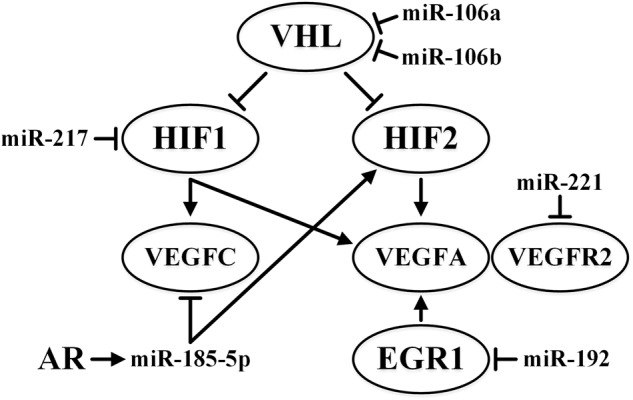
Summary of the effects of miR-106a, miR-106b, miR-185-5p, miR-192, miR-217, and miR-221 on VHL/HIF pathways in ccRCC. ↑ indicates activity stimulation and ⊤ indicates activity suppression according to any of the mechanisms.

The influence of some miRNAs on the VHL/HIF pathway is summarized in Figure 3.

In turn, the expression of certain miRNAs is affected by the proteins of the VHL-HIF-VEGF pathway (for example, Schanza et al., 2017). Thus, knockdown or genetic elimination of the *VHL* gene in RCC cells leads to an increase in miR-28-3p expression. The target of this miRNA is the gene for mitotic spindle checkpoint protein (Mad2), which blocks cells with unformed spindles from entering mitosis. Dysregulation of Mad2 expression leads to chromosomal instability (Hell et al., 2014). The expression of miR-204 in ccRCC cells is, on the contrary, induced by VHL. The target of miR-204 is *MAP1LC3B* (*LC3B*), which is associated with macro-autophagy (Mikhaylova et al., 2012). LC3B-mediated macro-autophagy is necessary for nutrient supply to the tumor during the period of metabolic stress and is important for cell survival, tumor growth, and increased aggressiveness (Sato et al., 2007). At the same time, hypoxia in RCC suppresses miR-30c under the influence of HIF (Huang J. et al., 2013).

As a result of the interaction of different proteins of the VHL/HIF/VEGF pathway, along with the participation of miRNAs, positive and negative feedback loops may appear that prolongs the initial effect of hypoxia.

In mice, renal ischemia/reperfusion procedure was reported to reduce the concentration of 40 miRNAs by almost half, while the concentration of 36 other miRNAs more than doubled (Liu et al., 2012). Quantitative RT-PCR performed 4–12 h after the procedure confirmed an increase in miR-210 concentration. In HUVEC-12 cells, angiogenesis is stimulated by increases in the expression of VEGF and VEGFR2, which are triggered by an increased miR-210 concentration (Liu et al., 2012). Since the target of miR-210 in chondrocytes and hepatocellular carcinoma cells is *HIF3A*, it is highly likely that this miRNA may regulate the switch between acute hypoxia and chronic hypoxia responses in these cells (Serocki et al., 2018). HIF1A may regulate miR-210 and has been shown to enhance its expression in RCC (Nakada et al., 2011). HIF1A-mediated enhancement of miR-210 expression was also shown in breast cancer, while HIF2A showed no regulatory effect (Camps et al., 2008). However, in 786-O RCC cells, HIF2A also influenced miR-210 expression (Zhang Z. et al., 2009). Increased expression of miR-210 is most typical for ccRCC (Valera et al., 2011).

A feedback loop has been constructed for the cardiomyocytes. HIF1 increases transcription of the MIR-21 gene, and miR-21, in turn, induces the expression of HIF1A via the regulation of PTEN/AKT signaling pathway (Liu et al., 2014). The expression of miR-21 is also increased in oral squamous cell carcinoma (OSCC). It is highly concentrated in the exosomes of OSCC cells and enhances the migratory and invasive properties of tumor cells in a mechanism that is dependent on HIF1A and HIF2A (Li et al., 2016).

During hypoxia, exosomes containing miR-135b are formed in multiple myeloma cells. Suppression of the HIF1AN by miR-135b in HUVEC cells leads to increased HIF1 activity and angiogenesis (Umezu et al., 2014). This mechanism remains turned on even after the restoration of the normal supply of oxygen, which prolongs the antihypoxic response.

Enhanced miR-18a expression after 24 h of hypoxia was observed in human choroidal endothelial cells (Han et al., 2015). Since HIF1A mRNA is the direct target of this miRNA, this interaction may act as the switch between the responses to acute hypoxia and chronic hypoxia (Han et al., 2015).

Hypoxia suppresses the expression of DICER, which is associated with miRNA processing. However, it is not understood how this does not lead to the total disruption of miRNA activity (Serocki et al., 2018). Some miRNAs, such as the tumor suppressor miR-182-5p, are more sensitive to hypoxic suppression of DICER expression than others. In addition to the aforementioned effect on the AKT pathway, miR-182-5p acts as an inhibitor of its target HIF2A, even though it is primarily manifest in VHL-deficient ccRCC cells (Fan Y. et al., 2016). The miR-200 family is another example of miRNAs that are sensitive to disruption of DICER expression. DICER is the direct target of miR-122, a miRNA that is highly expressed in ccRCC, while the loss of DICER decreases the expression of the miR-200 family, leading to EMT (Fan Y. et al., 2018).

Most tumor cells respond to hypoxia by shifting their energy metabolism to glycolysis (the Warburg Effect). Many miRNAs participate in this shift, which is a characteristic of many solid tumors (Morais et al., 2017). In particular, in RCC, miR-1291 directly inhibits the expression of the *GLUT1* glucose transporter gene, and its levels in tumor tissue are reduced as compared to the levels in normal tissue (Yamasaki et al., 2013). In ccRCC, hypoxia-induced miR-101 expression can be a mechanism for activating glycolysis, as miR-101 inhibits the *TIGAR* (TP53-Induced Glycolysis and Apoptosis Regulator) gene, which is a member of the p53 pathway that is involved in mediating the exchange to the pentose phosphate pathway (Xu et al., 2017). However, TIGAR-mediated regulation of glycolysis has an anti-apoptotic effect, protecting against DNA damage.

lncRNAs also affect the transition to glycolysis (Serocki et al., 2018). In cell cultures obtained from solid tumors, HIF1A-inducible long intergenic non-coding (linc) RNA-p21 was shown to bind to HIF1A, disrupting its interaction with VHL and attenuating the ubiquitination of HIF1A. As a result, both HIF1A and lincRNA-p21 accumulate that causes a transition to glycolysis (Yang et al., 2014).

## Metastasis and EMT in Kidney Cancer: the Role of miRNA

The incidence of metastasis is high in ccRCC (25–30%) and increases to over 50% after surgery (Rydzanicz et al., 2013). Metastatic ccRCC is extremely resistant to therapy, with responses to chemo-, radio-, and immunotherapy observed in no more than 10% of patients, with 5-year survival rates as low as 9% in the presence of distant metastases (Randall et al., 2014).

Metastasis of primary tumors occurs in several stages – from changes in biochemistry, morphology, and migration abilities of tumor cells to the appearance of surface receptors that provide directed migration to target organs, followed by the formation of specific microenvironment in the target organ, into which metastatic cells can enter and survive.

Epithelial-mesenchymal transition is an important stage in metastasis, during which changes occur in the properties of cancer cells. The changes contribute to metastasis. The process of EMT is characterized by a loss of cell polarity and intercellular bonds, as well as an increase in the migration activity of cells and their invasive properties. A critical event in EMT is the loss of E-cadherin and an increase in the activity of its transcriptional repressors, which include *ZEB1, ZEB2, TWIST, SNAIL*, and *SLUG* (Piva et al., 2016). EMT causes the development of sarcomatoid ccRCC (the most unfavorable form of cancer) due to the switched expression of E-cadherin to N-cadherin, dissociation of β-catenin from the membrane, and increased expression of *SNAIL* and *SPARC* (Conant et al., 2011).

An evaluation of the expression of 11 EMT markers in nephrectomized samples of RCC revealed that E-cadherin, clusterin, TWIST, and vimentin levels were significant predictors of recurrence (Harada et al., 2012).

Wilms’ tumor, WT1, causes mesenchymal-to-epithelial transition (MET) in kidneys during their development. During development of other organs, such as the heart, this gene causes EMT. WT1 is absent in normal renal tissue but is expressed in ccRCC due to the decreased expression of VHL or expression of mutated VHL. WT1 expression in ccRCC in the presence of SNAIL and E-cadherin starts a “hybrid” process in which EMT and MET traits are simultaneously expressed (Sampson et al., 2014). The loss of VHL activity also stimulates EMT, thereby increasing the expression of HIF1A, which subsequently activates nuclear factor-kappa B (NF-κB). The cytoplasmic expression of NF-kB has been correlated with the invasiveness of ccRCC (Kankaya et al., 2015).

Chronic oxidative stress-stimulated malignant transformation of kidney cells is accompanied by their acquisition of stem characteristics and EMT (Mahalingaiah et al., 2015). This leads to a significant increase in the expression of genes such as *BCL2, CCND1* (CyclinD1), *BIRC5* (Survivin), and *PCNA*, as well as *VIM* (Vimentin), *ACTA2* (α-Smooth muscle actin, *α-SMA*) and *SNAIL1*, as well as a significant decrease in the expression of *CDH1* (E-cadherin), cytokeratin, and *CTNNB1* (β-catenin*)*. The suppression of *OCT4* and *SNAIL* with small interfering RNA (siRNA) can partially reverse these changes (Mahalingaiah et al., 2015). The effect of hypoxia on EMT can also be partially achieved by reducing the expression of miR-30c and increasing the expression of its target, *SLUG*, which also leads to a decrease in E-cadherin expression and stimulation of cell migration in RCC (Huang J. et al., 2013).

Downregulation of *FOXO3a* in ccRCC also leads to an increase in expression of *SNAIL* that stimulates EMT (Ni et al., 2014). In ccRCC, *FOXO3a* is a target of oncogenic miR-155, which increases proliferation, colony formation, migration, and invasion (Ji et al., 2017). *SNAIL1* in ccRCC is the target of miR-30e-3p (minor variant), which inhibits invasion and migration of tumor cells (Wang D. et al., 2017).

The level of miR-720 is elevated in RCC, while its downregulation suppresses tumor growth (Bhat et al., 2017). miR-720 in RCC targets *CTNNA1* (αE-catenin) and *CDH1* (E-cadherin), thereby stimulating cell migration and invasiveness.

ZEB2 suppresses the expression of E-cadherin. In RCC, ZEB2 enhances the migratory and invasive ability of cells and the expression of ZEB2 correlates with more advanced forms of the disease and worse survival rates (see the review by Piva et al., 2016). One of the targets of miR-141 (a member of the miR-200 family) in RCC is *ZEB2* and thus it can suppress EMT (Li W. et al., 2014). Reduced expression of miR-141 has been correlated with a poor response to sunitinib (Berkers et al., 2013). In addition, in RCC, miR-141 binds to the lncRNA HOTAIR causing its cleavage by the Argonaute 2 (Ago2) complex (Chiyomaru et al., 2014). As already mentioned, HOTAIR is also capable of stimulating EMT (Hong et al., 2017). The high expression of miR-122 in ccRCC stimulates EMT as a result of the targeting of *DICER* and *OCLN* (occludin), which plays an important role in tight junctions, which are characteristic of renal tissue. The loss of tight junctions leads to an increase in cell mobility (Jingushi et al., 2017). Downregulated expression of DICER results in the suppression of the maturation of the miR-200 family.

In RCC, three miRNAs – miR-192 (which has anti-angiogenic effects), miR-194, and miR-215 – target *ZEB2, MDM2*, and *TYMS* oncogenes (Khella H.W. et al., 2013). miR-30a is specifically downregulated in metastatic tumors compared to its expression in primary tumors (Butz et al., 2015). Other studies on ccRCC have implicated the *ZEB2* and glucose-regulated protein 78 (*GRP78*) genes as targets of miR-30a (Chen Z. et al., 2017; Wang C. et al., 2017).

Expression of the transcription factor SOX4, which is normally involved in embryogenesis, is limited to a small number of cell types in which the mature cells retain stem cell characteristics. Due to the influence of SOX4 on the Wnt/β-catenin, Notch1, and p53 pathways, as well as the components of the miRNA processing machinery (DICER, Argonaute 1, RNA helicase A), upregulation of the expression of SOX4 can cause the development of different types of cancer and stimulate EMT. In contrast, the high expression of SOX4 may be associated with a more favorable disease course in other types of cancer. In different cancer types, many miRNAs target *SOX4*. They usually act in an anti-oncogenic manner and are characterized by reduced expression in ccRCC (Geethadevi et al., 2018). In ccRCC, miR-138 has a tumor suppressor role via the targeting of *SOX4*. The effects include the inhibited proliferation, migration, and invasion of tumor cells, increased expression of E-cadherin, and decreased expression of vimentin (Liu F. et al., 2017).

In ccRCC, miR-210 targets *TWIST1*. When the expression of this miRNA is turned off, the cancer cells begin to display the EMT morphology and increased tumor growth is observed in xenografts. High TWIST1 expression along with low expression of miR-210 is associated with a low survival rate in ccRCC. The aforementioned association of miR-210 with a response to hypoxia and its angiogenic behavior is important. It cannot be ruled out that miR-210 may show varied roles at different stages in the development of ccRCC (Yoshino et al., 2017).

**FIGURE 4:**
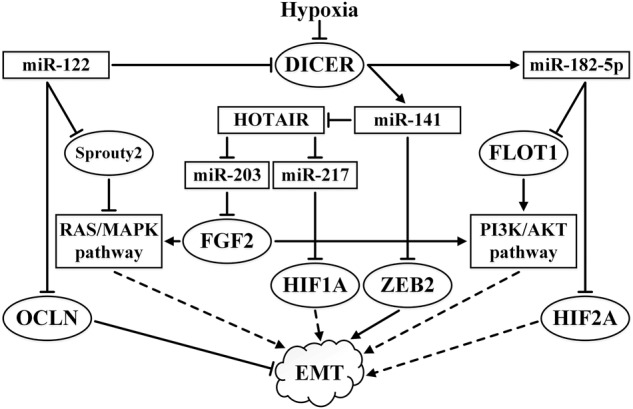
Summary of the effects of miR-122, miR-141, miR-182-5p, miR-203, and miR-217 and their interactions with lncRNA HOTAIR on EMT-related pathways in ccRCC. ↑ indicates activity stimulation and ⊤ indicates activity suppression according to any of the mechanisms. Solid lines indicate direct influence, while the dashed lines indicate an effect through intermediaries.

The reduced expression of miR-203 in ccRCC primary tumors correlates with poor prognosis and metastasis, while increased expression of miR-203 suppresses RCC cell lines growth and ccRCC metastasis (Xu M. et al., 2015). The authors attributed this to the action of miR-203 on fibroblast growth factor 2 (*FGF2*) as a target. The FGF2 protein is able to interact with the dimer of growth factor receptor-bound protein 2 (GRB2) involved in the PI3K/AKT and Ras/MAPK signaling pathways. Moreover, FGF2 is involved in the fine regulation of the Ras/MAPK pathway via negative feedback. FGF2 is overexpressed in many cancers (Ornitz and Itoh, 2015). The interaction between lncRNA HOTAIR and miR-203 leads to the suppression of miR-203 and stimulates EMT; when this interaction is eliminated, the expression of E-cadherin, claudin, PTEN, p21, and p27 increase, while vimentin expression is reduced (Hong et al., 2017). Methylation of MIR-203 is a specific characteristic of metastatic forms of ccRCC (see below and Pereyaslova et al., 2016). The influence of some miRNAs and lncRNA HOTAIR on EMT-related pathways is summarized in Figure 4.

The role of the cell environment, in particular the intercellular matrix, is important in metastasis. *CD44* is one of the important targets of miR-34a in the RCC lines. The CD44 protein is a cell surface glycoprotein that is involved in the intercellular interaction, cell adhesion, and migration. CD44 is a receptor for hyaluronic acid, osteopontin, collagen, and it can interact with matrix metalloproteinases. Ectopic expression of miR-34a stops cellular growth, migration, and invasion, and also significantly inhibits the growth of carcinoma in xenografts and metastasis in nude mice (Yu et al., 2014).

ADAM metallopeptidase domain 17 (*ADAM17*, also known as disintegrin and A metalloproteinase 17) is a target of miR-145 in RCC. The level of ADAM17 is significantly increased in RCC, whereas the level of miR-145 is reduced. ADAM17 stimulates cell proliferation and migration, and also negatively regulates miR-145 through tumor necrosis factor-alpha (TNFα). The result is the formation of a positive feedback loop. In healthy kidneys, this metalloproteinase is found in various types of cells capable of giving rise to RCC (Doberstein et al., 2013).

The expression of miR-186 is greatly reduced in RCC. This miRNA suppresses cell growth, colony formation, invasion, and arrests the cell cycle at the G0/G1 stage. It is believed that the effects of miR-186 are due to the action on *SENP1* (sentrin/SUMO-specific protease 1) as a target (Jiao et al., 2018). The SENP1 protein is the regulator of the SUMO pathway, which triggers the detachment of SUMO from homeodomain interacting protein kinase 2 (HIPK2, a transcriptional regulator), histone deacetylase 1 (HDAC1), and metastasis associated 1 (MTA1). The influence of the mimic miR-186 on *SENP1* can suppress the pathway of NF-kB, which reduces the expression of p-IkBa and p-p65, as well as the underlying cyclin D1 and MMP9, and increases the expression of p21 and BCL-2 (Jiao et al., 2018).

Reduced expressions of miR-149-5p and miR-149-3p are correlated in ccRCC with increased tumor aggressiveness and metastases (Okato et al., 2017). The authors attributed this to the fact that the target of both miRNAs is forkhead box protein M1 (*FOXM1*), a transcriptional activator that regulates the expression of cyclins, such as cyclin B1 and cyclin D1.

## Aberrantly Methylated miRNA Genes in ccRCC

One of the critical means of regulating the expression of miRNA genes is the methylation of the CpG island that is adjacent to or overlaps with the miRNA gene (Han et al., 2007; Lopez-Serra and Esteller, 2012). It is assumed that the percentage of miRNA genes that are aberrantly methylated in tumors is several times higher than the genes encoding proteins, which increases their prospects as biomarkers (Kunej et al., 2011; Baylin and Jones, 2016; Piletic and Kunej, 2016). Methylation profiles of miRNA genes have been constructed for epithelial tumors that have different localizations, including the colon, lung, breast, prostate, and ovaries. These are being considered as new potential markers and marker systems for the diagnosis and prognosis of these malignant cancers (Pronina et al., 2017b; Torres-Ferreira et al., 2017; Heller et al., 2018; Loginov et al., 2018a,b; Shi et al., 2018; Zare et al., 2018).

In contrast to the most common and widely studied types of cancer, such as colon, lung, breast, and prostate cancer, less is known about the methylation of the miRNA genes in ccRCC. What is known mainly concerns genes of the miR-9 and miR-34 families (Hildebrandt et al., 2010; Vogt et al., 2011; Schiffgen et al., 2013). These studies have shown that the promoter of the gene coding for miR-34a is significantly methylated in RCC cell lines and that the expression of this miRNA is downregulated. Treatment with 5-aza-2′-deoxycytidine increases the expression of miR-34a and reduces the expression of its target (Yu et al., 2014).

The last 5 years have seen a significant increase in the understanding of the role of hypermethylation of miRNA genes in the pathogenesis of ccRCC. In ccRCC, miR-10b is characterized by a highly methylated promoter and significantly reduced expression (He C. et al., 2015). Transfection by lentivirus carrying this miRNA or treatment with demethylating agents inhibits cell proliferation, migration, and invasiveness in cell culture. However, the target genes that are significant in RCC are unknown (He C. et al., 2015). In stomach cancer, miR-10b acts as a tumor suppressor and targets microtubule-associated protein RP/EB family member 1 (*MAPRE1*) (Kim et al., 2011). In non-small-cell lung cancer and bladder cancer, it acts as an oncogene by enhancing cellular migration and invasion. The putative targets associated with this effect are *KLF4* and *HOXD10* (Xiao et al., 2014).

A high miR-21/miR-10b ratio in metastatic ccRCC correlates with a poor prognosis (Fritz et al., 2014). Interestingly, an increased level of MIR-21 expression that correlates with a poor prognosis is associated with hypomethylation of its promoter (Cancer Genome Atlas Research, 2013).

The aforementioned miR-182-5p tumor suppressor miRNA can also be regulated by methylation. An upstream CpG island (8–10 kb) contains a putative transcription start site. Its methylation level is significantly increased in RCC cell cultures and is slightly elevated in ccRCC samples compared to the normal tissue, which may explain the decreased expression of this miRNA. One of the targets of miR-182-5p is *MALAT-1*. This gene produces a precursor transcript from which a ncRNA is derived by RNase P cleavage of a tRNA-like small ncRNA (known as mascRNA) from its 3′ end. Its ribonucleoprotein complexes may act as a transcriptional regulator for numerous genes, including some genes involved in cancer metastasis and cell migration. Downregulation of *MALAT-1* leads to upregulation of p53 and downregulation of CDC20 and AURKA, which are the drivers of the cell cycle mitotic phase (Kulkarni et al., 2018).

The gene encoding miR-766-3p is highly methylated in RCC tissues compared to the normal tissues. This miRNA behaves like a suppressor. Its direct target is *SF2*, whose repression also reduces the expression of AKT and ERK (Chen C. et al., 2017). The promoter of miR-145, which targets the metallopeptidase gene *ADAM17*, is also strongly methylated in RCC. Treatment with 5-aza-2′-deoxycytidine increases the expression of miR-145 and reduces the expression of *ADAM17* (Doberstein et al., 2013). The promoter region of the MIR-200c gene is hypermethylated in RCC cell lines, which correlates with decreased expression, and it is not methylated in the normal renal cell line (Gao et al., 2014). The MIR-492 gene has a strongly methylated promoter in ccRCC and reduced expression. The use of 5-aza-2′-deoxycytidine or the histone deacetylase inhibitor 4-phenylbutyric acid increases the expression of miR-492 in ccRCC cell cultures, inhibits cell proliferation, and stimulates apoptosis and adhesion (Wu et al., 2015). The upstream promoter of MIR-106a gene is hypermethylated in RCC, which might be responsible for its downregulation (Pan et al., 2017).

Over the past 5 years, we have systematically analyzed the methylation of up to 20 miRNA genes in various cancers, including lung, breast, ovarian, and kidney cancer. These analyses have identified new markers and marker systems that are useful for the diagnosis and prognosis of these malignant diseases (Beresneva et al., 2013; Rykov et al., 2013; Pronina et al., 2017b; Braga et al., 2018; Loginov et al., 2018a,b; Varachev et al., 2018). Moreover, we have shown a correlation between the levels of expression and methylation, which has confirmed the functional role of methylation of a group of miRNA genes in the pathogenesis of breast, ovarian, and kidney cancer (Pronina et al., 2017b; Loginov et al., 2018a,b; Varachev et al., 2018).

We first found that the frequency of methylation of six miRNA genes (MIR-124-2, MIR-124-3, MIR-9-1, MIR-9-3, MIR-34b/c, and MIR-129-2) was significantly higher in malignant tumors of patients with ccRCC than in normal kidney tissues (Beresneva et al., 2013). Subsequent studies by our group established that 16 miRNA genes (MIR-124-1/-2/-3, -125b-1, MIR-129-2, MIR-132, MIR-137, MIR-193a, MIR-34b/c, MIR-375, MIR-203, MIR-9-1, MIR-9-3, MIR-107, MIR-130b, and MIR-1258) were hypermethylated and two miRNA genes (MIR-191, MIR-212) were hypomethylated in ccRCC (Pereyaslova et al., 2016; Loginov et al., 2017; Varachev et al., 2018). Moreover, seven miRNA genes (MIR-124-3, MIR-125b-1, MIR-129-2, MIR-137, MIR-34b/c, MIR-375, MIR-9-3) were downregulated and correlated with altered methylation. The findings provided evidence of the functional significance of methylation in the deregulation of miRNA genes and in the pathogenesis of ccRCC (Varachev et al., 2018).

Besides hypermethylation, some miRNA genes studied (MIR-124-2/-3, -34b/c, MIR-129-2, MIR-107, MIR-148a, MIR-203) have been associated with aspects of ccRCC progression that include advanced pathologic stage, tumor size, and differentiation grade. In particular, hypermethylation of six miRNA genes (MIR-125b-1, MIR-129-2, MIR-203, MIR-375, MIR-107, and MIR-1258) significantly correlated with the presence of metastasis (Pereyaslova et al., 2016; Varachev et al., 2018), and most significantly for MIR-375 and MIR-1258 (*p*-value ∼ 10^-7^–10^-8^).

On the basis of hypermethylated miRNA genes, novel marker systems have been suggested for ccRCC diagnosis (MIR-125b-1, MIR-375, MIR-137, MIR-193a) and prediction of metastasis (MIR-125b-1, MIR-375, MIR-107, MIR-1258, MIR-203) (Loginov et al., 2017; Varachev et al., 2018).

A Russian team of researchers demonstrated the correlation of the methylation of the MIR-129-2 gene with increased expression of genes, such as *RARB(2), RHOA, NKIRAS1*, and *CHL1* (Pronina et al., 2017a). Preliminary data from another study indicated a negative correlation between the expression levels of miR-375 and the pro-apoptotic gene *APAF1* (Varachev et al., 2018). The targets of most hypermethylated miRNAs, identified by us recently remain unclear and further research is needed.

However, the roles of some of the novel hypermethylated miRNAs in the pathogenesis of kidney cancer have been studied. For instance, the reduced expression of miR-124 is considered a highly significant factor in ccRCC genesis and may be an essential predictor of survival (Butz et al., 2015). Moreover, the miRNA and gene expression data from 458 ccRCC and 254 normal kidney specimens were used to construct the integrated miRNA-target interaction network, which revealed miR-124 as a key miRNA contributing to the aggressive behavior of ccRCC (Butz et al., 2015).

Table 1 presents examples of miRNAs encoded by genes that are hypermethylated and downregulated in ccRCC. Data on target genes and functions in RCC are also provided. The information presented pertains mainly to those genes whose role of hypermethylation was first explored by our group and data from other authors concerning the functional roles, target genes, and in some cases the signaling pathways involved.

**Table 1:** Examples of the most studied miRNAs encoded by hypermethylated genes in ccRCC, focusing on the role of aberrant methylation in miRNA deregulation, target genes, and functions.

miRNA	miRNA gene hypermethylation and downregulation	Target genes of miRNA and some pathways	Functions of miRNA in ccRCC	References
miR-124-3p	Hypermethylation of the *miR-124-2* gene correlates with decrease of miR-124-3p levels in ccRCC tissues and cell lines	*CAV1, FLOT, FZD5* (miR-124-3p/FZD5/PKC pathway), lncRNA HOTAIR, *ST8SIA4* (HOTAIR/miR-124/ST8SIA4 pathway)	Hypermethylation of *miR-124-2* is strongly associated with advanced pathologic stage and grade of differentiation. Restoration miR-124-3p reduces migration, invasion, proliferation, metastasis; decreased cell cycle S phase; increases patient survival, drug resistance. By sponging miR-124 HOTAIR as ceRNA upregulated ST8SIA4 and promoted the proliferation and metastasis in RCC	Butz et al., 2015; Long et al., 2015; Pan et al., 2018b; Varachev et al., 2018
miR-129-3p	Hypermethylation of the *miR-129-2* gene correlates with decrease of miR-129-3p levels in ccRCC tissues and cell lines	*SOX-4, MMP-2/9, TRPM7* (miR-129-3p/TRPM7/AKT/FOXO1 pathway)	Hypermethylation of *miR-129-2* is associated with advanced pathologic stage and metastasis in ccRCC; miR-129-3p inhibits migration, invasion, metastasis	Beresneva et al., 2013; Chen et al., 2014; Zhao Z. et al., 2018
miR-137	Downregulation of miR-137 in RCC tissues and cell lines correlates with hypermethylation of the *miR-137* gene	lncRNA SNHG1, oncogene *RLIP76*	Decrease proliferation, migration, invasion, metastasis; promotes apoptosis; inhibits PI3K/PKB pathway; tumor suppressor function confirmed *in vitro* and *in vivo*	Zhang and Li, 2016; Varachev et al., 2018; Wang M. et al., 2018; Zhao S. et al., 2018
miR-182-5p	Downregulation correlates with hypermethylation in RCC tissues and cell lines	HIF-2α, lncRNA MALAT-1, *FLOT1*	Oncosuppressor; inhibited tumorigenicity *in vitro* and *in vivo* and induced mitotic cell cycle arrest and apoptosis	Xu et al., 2014; Fan Y. et al., 2016; Fang et al., 2018; Kulkarni et al., 2018
miR-203	Downregulation of miR-203 in RCC cell lines and ccRCC specimens; methylation of the *miR-203* gene is significantly increased only in metastatic tumors	*FGF2*, lncRNA SNHG14, N-WASP (SNHG14/miR-203/N-WASP pathway), lncRNA HOTAIR	*miR-203* methylation and low expression are associated with metastasis in ccRCC. SNHG14 sponging miR-203 and elevating N-WASP as ceRNA promotes ccRCC migration and invasion. Overexpression of miR-203 inhibited migration, invasion, and induced apoptosis and cell cycle arrest. miR-203-HOTAIR interaction inhibited EMT and metastatic genes	Xu M. et al., 2015; Liu G. et al., 2017; Dasgupta et al., 2018; Varachev et al., 2018
miR-375	Downregulation in RCC cell lines and ccRCC specimens; the *miR-375* gene is hypermethylated in ccRCC tissues	Oncogene *YWHAZ*	*miR-375* methylation and low expression are associated with metastasis ccRCC; according to functional studies miR-375 suppressed ccRCC cell proliferation, migration, and invasion	Varachev et al., 2018; Zhang X. et al., 2018
miR-766-3p	Downregulation caused by hypermethylation in RCC tissues	SF2 (miR-766-3p/SF2/P-AKT/P-ERK pathway)	Downregulation is associated with clinical stage and worse prognosis; upregulation attenuates cell cycle progression	Chen C. et al., 2017

For five miRNAs we identified (miR-124-3p, miR-129-3p, miR-137, miR-203, and miR-375), functional studies by other authors confirmed suppressor properties and/or association with metastasis. The tumor suppressor and anti-metastatic functions of the hypermethylated MIR-129-2 gene we discovered (Beresneva et al., 2013), are consistent with the data that miR-129-3p downregulates multiple metastasis-related genes in RCC cells, including *SOX4*, and also decreased phosphorylation of focal adhesion kinase and expression of *MMP-2/9* (Chen et al., 2014). In addition, miR-129-3p is involved in miR-129-3p/TRPM7/AKT/FOXO1 signaling pathway (Zhao Z. et al., 2018). The collective data confirm the ability of miR-129-3p to restrain metastasis in ccRCC.

In ccRCC, the hypermethylated MIR-137 gene (Varachev et al., 2018) abrogates the tumor-promoting effect of overexpressed ncRNA Small Nucleolar RNA Host Gene 1 (SNHG1), which is associated with metastasis and poor prognosis of ccRCC patients (Zhao S. et al., 2018).

Data on the nature of the role of miR-203 in RCC, either as a tumor suppressor or an oncogene, is inconsistent (Hu et al., 2014). However, in other cancer types (hepatocellular carcinoma, multiple myeloma, lung cancer, esophageal cancer, bladder cancer), miR-203 acts as a suppressor (Hu et al., 2014). It is possible that this miRNA has different roles at different stages of the development of ccRCC and/or in its different variants. Overall, we observed significantly more frequent hypermethylation of the MIR-203 gene in tumors of patients with ccRCC with metastases. Moreover, our data on the anti-metastatic activity of miR-203 are consistent with data from three other papers (see Table 1), including the results on the role of miRNA-203 in inhibition of EMT and metastatic genes (Dasgupta et al., 2018).

Our data concerning the correlation of miR-375 hypermethylation with metastasis strengthens the evidence that the suppression of the tumor aggressive phenotypes of ccRCC mediated by this miRNA regulates the oncogene *YWHAZ*, which is its direct target (Zhang X. et al., 2018). As discussed earlier, aberrantly methylated miR-148a was suggested to function as a tumor suppressor in RCC by targeting *AKT2* (Cao et al., 2017). A recent paper (Reustle et al., 2018) implicated the role of aberrantly methylated miR-212-3p and miR-132-3p in ccRCC in the post-transcriptional regulation of *BCRP/ABCG2* (breast cancer resistance protein), which contributes to the multi-drug resistance seen in cancer. Hypermethylated miR-193a-3p can affect the PI3K/AKT pathway in RCC (Pan et al., 2018a).

However, our data on the frequent hypermethylation of the MIR-125b-1 gene and the hypermethylation linkages with progression and metastasis (Varachev et al., 2018) do not agree with the increased level of this miRNA and its role in promoting cell (Jin et al., 2017). The properties of this miRNA are also ambiguous in other types of cancer, such as breast cancer (Loginov et al., 2018a). Additional studies are required to determine the role and mechanism of miR-125b in RCC.

Tumor suppressor or/and anti-metastatic properties were found for some miRNAs, encoded by 16 aberrantly methylated genes that discovered by us, including miR-124, miR-129, miR-132, miR-137, miR-148a, miR-203, and miR-375, as well as for miR-9 and miR-34 genes. However, further analyses should explore the expression and functional properties of some other miRNAs, which genes were also found as hypermethylated (miR-107, miR-125b, miIR-1258, miR-130b, and miR-193a) or showed hypomethylation (miR-191, miR-212) in cancers of the kidney. Overall, our group has provided novel data on the contribution of methylation to the regulation of more than 10 miRNA genes in ccRCC (Beresneva et al., 2013; Loginov et al., 2017; Varachev et al., 2018). Further research and validation of target genes for these aberrantly methylated miRNAs are required. Moreover, hypermethylated miRNAs could someday be used as convenient markers for ccRCC in the clinic, as has been suggested for non-invasive diagnosis and prognosis of prostate cancer using miR-193b, miR-129-2, and miR-34b/c methylation in tissue and urine samples (Torres-Ferreira et al., 2017).

In Table 1, it is interesting to note that among the seven most studied hypermethylated miRNAs, four have lncRNAs as one or more targets. For example, miR-124-3p interacts with the lncRNA HOTAIR, miR-137 with lncRNA SNHG1, miR-182-5p with lncRNA MALAT1, and miR-203 with the HOTAIR and SNHG14 lncRNAs. These data reinforce the role of lncRNAs in the regulatory functions of miRNA, apparently by acting primarily as ceRNA to reduce the content of specific miRNAs depending on other factors.

## Clinical Application of miRNAs as Markers for Diagnosis and Prognosis

In this review, we included only the most convincing results obtained from studies featuring large sample sizes and detailed statistical evaluations. Initially, we examined the features of miRNAs that are relevant concerning their diagnostic potential. These features are summarized in Table 2.

**Table 2:** Non-invasive markers suggested for use in ccRCC diagnosis: miRNA level and diagnostic value.

miRNA and level	Diagnostic value	Type and size of samples used	References
miR-378 ↑, miR-451 ↓	AUC 0.86, Sn 81%, Sp 83%	15 ccRCC + 90 RCC (73 ccRCC), 12+35 healthy controls	Redova et al., 2012
miR-210 ↑	AUC 0.81, Sn 74%, Sp 76%	Meta-analysis – 570 RCC patients	Chen et al., 2018
miR-378 ↑, miR-210 ↑	Sn 80%, Sp 78%	157 ccRCC; 12 chromophobe RCC; 26 papillary RCC	Fedorko et al., 2015
miR-193a-3p ↑, miR-362 ↑, miR-572 ↑, miR-28-5p ↓, miR-378 ↓	AUC 0.81 – for training sample set; AUC 0.80 – for the validating sample set	25+107 ccRCC patients	Wang et al., 2015
miR-210 ↑	Sn 70%, Sp 62%	82 ccRCC patients, serum exosomes	Zhang W. et al., 2018
miR-1233 ↑	Sn 81%, Sp 76%		
miR-34a ↓	Sn 81%, Sp 80%	30 ccRCC patients	Yadav et al., 2017
miR-141 ↓	Sn 75%, Sp 73%		
miR-1233 ↑	Sn 93%, Sp 100%		
miR-141 ↓, miR-1233 ↑	Sn 100%, Sp 73%.		
miR-144-3p ↑	AUC 0.91, Sn 87%, Sp 83% – ccRCC vs healthy donors; AUC 0.82, Sn 75%, Sp 72% – ccRCC vs renal angiomyolipomas	106 ccRCC, 28 renal angiomyolipomas, 123 healthy donors	Lou et al., 2017

*Note: ↓ – decreased level of miRNA; ↑ – increased level of miRNA; AUC – area under the receiver operating characteristic curve, Sn – sensitivity, Sp – specificity. Only the most convincing results were included, with sample sizes ≥ 30 and detailed statistical evaluations: AUC, sensitivity, and specificity.*

The diagnostic value of elevated miR-210 levels is well established. This may be due to chronic hypoxia observed in ccRCC (Zhang Z. et al., 2009). However, no correlation of miR-210 expression with tumor stage has been reported (Iwamoto et al., 2014). The expression of miR-1233 in RCC cell lines is also stimulated by hypoxia (Dias et al., 2017).

The target of miR-451 is an mRNA encoding the PSMB8 protein, which has a pro-inflammatory function, and which is presumably significant in RCC (Zhu et al., 2016). In RCC, miR-193a-3p affects the PI3K/AKT pathway (Liu L. et al., 2017; Pan et al., 2018a). miR-28-5p is associated with chromosomal instability (Hell et al., 2014). In addition, its target is Ras-related small GTP-binding oncoprotein RAP1B (Wang et al., 2016). miR-141 is involved in the suppression of EMT (Li W. et al., 2014).

Targets of miR-144 include *mTOR* (Xiang et al., 2016) and *MAP3K8* (Liu F. et al., 2016). Thus, miR-144 can suppress cellular proliferation, EMT, and metastasis. However, another study (Xiao et al., 2017) implicated miR-144 as having an oncogenic role, with involvements in the stimulation of proliferation, migration, invasion, and resistance to sunitinib. Furthermore, miR-144 acts as an upregulated marker in the plasma of ccRCC patients, particularly those with advanced pT stage, which is a characteristic of oncogenes (Lou et al., 2017). The inconsistent functional properties of miR-144 in ccRCC revealed to date highlight the need for further studies before its clinical application can be realized.

miR-378 stands apart from other miRNAs. In a study that used samples from patients at all stages of ccRCC (Redova et al., 2012), the diagnostic value of the increased expression of miR-378 was found. However, when samples were used only from patients at the I-II stage, the decreased expression of miR-378 had diagnostic value (Wang et al., 2015). This dichotomy of miRNA – with an oncogenic role in some types of cancer or at certain stages of the same disease, while being a tumor suppressor in other cases – is quite common. In ccRCC, miR-378 could be a potential marker of the disease stage (Li H.C. et al., 2014; Fedorko et al., 2015). However, Hauser et al. (2012) rejected the correlation between its expression in serum and pathologic stage. The authors also did not find evidence of differential expression of miR-378 levels in diseased and healthy individuals. The expression of miR-378 is likely associated with angiogenesis (Li H.C. et al., 2014). Indeed, in the ccRCC cell line, the expression of miR-378 declines with prolonged hypoxia (Chen S.C. et al., 2017). However, the mechanisms of action of miR-378 in ccRCC have not yet been adequately studied.

The genes discussed above, including some with inconsistent features (miR-144 and miR-378), have been suggested for the diagnosis of ccRCC (Table 2). Thus, the methods of ccRCC diagnosis need further verification.

The level of some miRNAs can be valuable as prognostic markers of survival in ccRCC (Table 3). Reviews describing miRNA markers used to predict survival and metastasis in ccRCC, have been published (Fedorko et al., 2016; Ran et al., 2017; He Y.H. et al., 2018). In the present review, we present only the most recent studies that featured large sample sizes (Table 3). Unfortunately, the data obtained so far has not always been consistent. For example, an increase of miR-210, which serves as a diagnostic criterion for ccRCC, was associated with prolonged overall survival in one study (McCormick et al., 2013) and decreased overall survival in another study (Samaan et al., 2015). For most miRNAs, the nature of interactions in ccRCC is not well understood.

**Table 3:** Prognostic markers for poor survival and metastasis in ccRCC: miRNA level and predictive value.

miRNA and level	Predictive value	References
miR-210 ↑	Lower stage and grade, mir-210 ↓ is associated with the worst OS	McCormick et al., 2013
miR-21 ↑, miR-126 ↓	Shorter CSS	Vergho et al., 2014
(miR-21/miR-10b)^∗^ ↑	Poor prognosis in M0	Fritz et al., 2014
miR-100 ↑	Worse OS and CSS	Wang et al., 2013
miR-155 ↓	Poor prognosis in stage III and IV (43 from 57 patients)	Shinmei et al., 2013
miR-217 ↓	Higher stage and grade, worse OS	Li H. et al., 2013
miR-497 ↓	Shorter OS	Zhao et al., 2015
miR-125b ↑	Worse CSS, early recurrence	Fu et al., 2014
miR-514 ↓	Primary M1 and recurrent	Wotschofsky et al., 2013
miR-204, miR-30c, miR-30a-3p, miR-30a-5p, miR-30e-3p, miR-30e-5p, miR-30c-2-3p ↓	M1 and distant metastasis vs M0	Heinzelmann et al., 2014
miR-30c, miR-126, miR-451 ↑	Shorter PFS and CSS	Heinzelmann et al., 2014
miR-215 ↓	Reduced DFS	Khella H.W. et al., 2013
miR-30a ↓	Hematogenous metastasis	Huang Q.B. et al., 2013
miR-630 ↑	Worst OS, metastasis	Zhao et al., 2014
miRNA-23b/27b cluster ↓	Worst OS, higher chance of disease recurrence	Ishihara et al., 2014
miR-29b ↑	Worst OS	Xu Y. et al., 2015
miR-126 ↓	Worst OS for large tumors (>4 cm)	Khella H.W. et al., 2015
miR-203 ↓	Worst OS; FGF2 – target (mitogenic and angiogenic activities)	Xu M. et al., 2015
miR-21, miR-1260b, miR-210, miR-100, miR-125b, miR-221, miR-630, miR-497 ↑; miR-106b, miR-99a, miR-1826, miR-215, miR-217, miR-187, miR-129-3p, miR-23b, miR-27b, miR-126 ↓	miR-21 ↑ is associated with the worst OS, CSS, DFS; miR-126 ↓ is associated with the worst OS, CSS, DFS	Gu et al., 2015
miR-21 ↑, miR-210 ↑, miR-141 ↓, miR-200c ↓, miR-429 ↓	Using these 5 miRNAs, a classificator of the risk of recurrence was constructed	Tang and Xu, 2015
miR-210 ↑	Worst OS, higher chance of disease recurrence	Samaan et al., 2015
miR-194 ↓	Worst OS and DFS	Nofech-Mozes et al., 2016
miR-429 ↓	Association with metastases and shorter DFS and OS. Prevents a reduction in the level of E-cadherin in EMT, reduces motility	Machackova et al., 2016
miR-10a-5p ↓	Association with tumor progression. If in the prediction of survival by stage and degree data use miR-10a-5p, AUC increased from 0.942 up to 0.995	Kowalik et al., 2017
miR-10b ↓	Level of miR-10b is significantly lower in primary ccRCC vs normal tissues; it is lower in primary ccRCC vs oncocytoma; it is lower in metastatic ccRCC vs primary ccRCC; it is lower in stages III/IV vs I/II. Decrease in expression is associated with smaller DFS and OS	Khella et al., 2017
miR-224 ↑	Correlation with significantly worse survival	Fujii et al., 2017
miR-18a-5p ↑	Correlation with significantly worse survival	Zhou et al., 2018
miR-122-5p ↓ miR-206 ↓ in ccRCC and in benign renal tumors	miR-122-5p ↑ correlates with metastases and worse survival; miR-206 ↑ correlates with pT stage and metastases and worse survival	Heinemann et al., 2018
miR-21 ↑, miR-142-5p ↑, miR-194 ↓	Correlation with metastases is characterized by 87% sensitivity and 82% specificity	Lokeshwar et al., 2018

*Note: ↓ – decreased level of miRNA; ↑ – increased level of miRNA; OS – overall survival; DFS – disease-free survival; CSS – cancer-specific survival; PFS – progression-free survival, ^∗^ – ratio. Only the most recent (since 2013) works in which sets of >35 ccRCC samples were included.*

Despite the relative simplicity of miRNA gene methylation studies, the approach has barely been used instead of the expression levels of miRNAs, to evaluate the prognosis of survival or predict metastasis in ccRCC. The only exception is a study (Gebauer et al., 2013) that described that the methylation of the gene encoding miR-124-3 was associated with a more advanced RCC stage, metastasis, and an increased risk of relapse in 111 RCC samples (80 ccRCC, with 77 paired with histologically normal tissue). Also, a recent review did not mention studies involving miRNA gene methylation in RCC (Ran et al., 2017). Studies we undertook identified novel biomarker systems by using hypermethylated miRNA genes as an indicator in ccRCC, both for the diagnosis (MIR-125b-1, MIR-375, MIR-137, and MIR-193a) and for the prediction of metastasis (MIR-125b-1, MIR-375, MIR-107, MIR-1258, and MIR-203) (Loginov et al., 2017; Varachev et al., 2018). The use of both biomarker systems in 70 patients was characterized by a high discrimination value [area under the curve (*AUC*) 0.93], sensitivity (86%), and specificity (95%).

Few studies have explored the use of the miRNA level as an indicator to predict the response to ccRCC treatment. Most of the studies have focused on predicting the success of the response to tyrosine kinase inhibitors that are the first-line treatment for metastatic ccRCC. However, approximately 20% of the patients who are treated rapidly develop resistance to these drugs. For example, for 74 patients with metastatic ccRCC who were treated with tyrosine kinase inhibitors, early progression of the disease was observed in 16 (Garcia-Donas et al., 2016). Selection of increased miR-1307-3p and miR-425-5p levels as the biomarker predicted the risk of poor outcome to treatment with an AUC value of 0.75, which is better than the risks predicted using parameters based on clinicopathological factors. This result was revalidated in a group of 64 patients (Garcia-Donas et al., 2016). The predictors of the outcome of sunitinib treatment were studied in 123 patients with ccRCC (the dataset included metastatic and non-metastatic ccRCC patients). Of the 123 patients, 97 were characterized by prolonged (>22 months) progression-free survival (PFS) and the remaining 26 showed rapid progression of the disease in the first 3 months (Puente et al., 2017). It must be noted that, along with the other characteristic clinical indices, the former group was distinguished by the presence of high levels of the transcription factors HEYL, HEY, and HES, accompanied by elevated levels of miR-27b, miR-23b, and miR-628-5p, although miR-23b is considered as a typical oncogene (Zaman et al., 2012). In another study, 56 sunitinib-treated patients with metastatic ccRCC (24 responsive patients with PFS > 18 months and 32 non-responsive patients with PFS < 6 months) (Kovacova et al., 2018). A good response to the drug was positively associated with miR-942 and miR-133 levels, and the model based on using their expression levels as indicators gave predictions with an AUC value of 0.81.

Another aspect of the use of miRNA as biomarkers is the predictive capabilities of miRNA polymorphisms (variations or polymorphisms in sequences of miRNA, target mRNA, miRNA genes, and target miRNA genes and their pathways). The polymorphism in the MIR-34b/c promoter region has been amply studied. This polymorphism reduces the expression of this miRNA (allele C for rs4938723) in the homozygotic condition, increases the risk of RCC in the Chinese population, especially in elderly, men, smokers, and alcohol abusers (Zhang S. et al., 2014). Polymorphisms in the miRNA-binding sites of 102 genes belonging to the VHL-HIF1α pathway and their effects on RCC risk have been analyzed (Wei et al., 2014). The most significant effect of two polymorphisms in the *MAPK1* gene, and the presence of four of the five adverse alleles in the *MAPK1, CDCP1, TFRC*, and *DEC1* gene set, was a more than twofold increase in the risk of RCC.

The genotype CC, a variant of the 3′ untranslated region (UTR) of the SET domain containing lysine methyltransferase 8 (*SET8*) gene, significantly reduced the risk of ccRCC (the odds ratio = 0.318). This single nucleotide polymorphism, also designated rs16917496, is located in the miR-502 binding site within the 3′UTR of the *SET8* gene and is associated with its downregulation in ccRCC. Knockdown of *SET8* inhibited proliferation, migration, and invasiveness of ccRCC cells in culture (Zhang et al., 2017). Many ccRCC-influencing genes are associated with remodeling, and *SET8* may be one of them (Joosten et al., 2018).

## Conclusion

In recent years, the role of miRNA in the pathogenesis of ccRCC has been amply studied. In ccRCC, miRNAs involved in processes that include the response to hypoxia, EMT, and chromatin remodeling play significant roles. Nevertheless, the picture is far from complete. The search for new diagnostic and prognostic markers is still largely empirical and the associated pathways and processes are not clearly elucidated. For example, in a study involving prediction and correlation analyses, miRNA–mRNA pairs displayed a significant inverse relationship. miR-30b was one of these miRNAs, and its expression was negatively correlated with the highest number of genes in ccRCC (Liu et al., 2018). Among the latter, there are, for example, genes that are significant for tumor invasion, such as Integrin Subunit Alpha 5 (*ITGA5*). Nevertheless, almost nothing is known concerning the influence of miR-30b on ccRCC. The many potential biomarkers include some differentially expressed miRNAs or differentially methylated sites. However, only a few have been experimentally verified (Wang Z. et al., 2018).

For the diagnosis of ccRCC, the use of miRNAs like miR-144 has been proposed since their targets may be relevant for this cancer. However, this use is hampered by inconsistent findings, with both tumor suppressive and oncogenic activity reported in separate studies. Results of various studies for other miRNAs, including miR-106a, miR-125b, miR-203, and miR-378, have been contradictory. These discrepancies may be due to the varying roles of these miRNAs at different stages of cancer or in different forms of the disease or to their ability to interact with various targets depending on the cellular context.

Our recent contributions have revealed the potential for the use of the methylation profiles of miRNA genes in the search for reliable diagnostic and prognostic marker systems (i.e., high AUC). The results have suggested the need to expand the search for the selection of new miRNA genes regulated by methylation. Moreover, in-depth studies of the identified hypermethylated miRNA genes associated with metastasis with respect to their target genes and functions are necessary.

The recently discovered lncRNAs represent a novel class of potential prognostic biomarkers in ccRCC. Data on the function and clinical applications of lncRNA in ccRCC is accumulating, with 60 articles identified in PubMed as of January 2019. However, only a few of these studies (10%) explored the interactions of lncRNAs with miRNAs. Nevertheless, a detailed analysis of the molecular mechanisms of ccRCC in original articles allowed us to identify at least seven miRNAs (miR-122, miR-124-3p, miR-137, miR-141, miR-182-5p, miR-203, and miR-217) that interact with the lncRNAs (e.g., HOTAIR, MALAT-1, SNHG1, and SNHG14). Further research is needed to elucidate these interactions in ccRCC and to assess their clinical potential.

## Author Contributions

EB, MF, VL, AD, and SM wrote the manuscript. All the authors revised the work critically for important intellectual content, approved the version to be published, and agreed to be accountable for all aspects of the work in ensuring that questions related to the accuracy or integrity of any part of the work are appropriately investigated and resolved.

## Conflict of Interest Statement

The authors declare that the research was conducted in the absence of any commercial or financial relationships that could be construed as a potential conflict of interest.
